# Adipokines, diabetes and atherosclerosis: an inflammatory association

**DOI:** 10.3389/fphys.2015.00304

**Published:** 2015-11-03

**Authors:** Leandro C. Freitas Lima, Valdir de Andrade Braga, Maria do Socorro de França Silva, Josiane de Campos Cruz, Sérgio H. Sousa Santos, Matheus M. de Oliveira Monteiro, Camille de Moura Balarini

**Affiliations:** ^1^Biological Sciences Institute, Federal University of Minas GeraisBelo Horizonte, Brazil; ^2^Biotechnology Center, Federal University of Paraiba (Universidade Federal da Paraíba)Joao Pessoa, Brazil; ^3^Health Science Post-Graduate Program, State University of Montes ClarosMontes Claros, Brazil; ^4^Health Sciences Center, Federal University of Paraiba (Universidade Federal da Paraíba)Joao Pessoa, Brazil

**Keywords:** adipokines, diabetes, atherosclerosis, adiponectin, TNFα, IL-6, MCP-1, leptin

## Abstract

Cardiovascular diseases can be considered the most important cause of death in diabetic population and diabetes can in turn increase the risk of cardiovascular events. Inflammation process is currently recognized as responsible for the development and maintenance of diverse chronic diseases, including diabetes and atherosclerosis. Considering that adipose tissue is an important source of adipokines, which may present anti and proinflammatory effects, the aim of this review is to explore the role of the main adipokines in the pathophysiology of diabetes and atherosclerosis, highlighting the therapeutic options that could arise from the manipulation of these signaling pathways both in humans and in translational models.

## Introduction

Diabetes mellitus (DM) is characterized by insufficient production of insulin (type 1) or, more commonly, inefficient insulin signaling pathways (type 2), a state known as insulin resistance (IR) (International Diabetes Federation, 2013; American Diabetes Association, 2014). Cardiovascular diseases (CVD) are the most important cause of death in the diabetic population (Skyler et al., 2009; American Diabetes Association, 2014). Obesity, a global health problem, is characterized by overproduction of inflammatory adipokines by adipose tissue and this may be the link between obesity, CVD and diabetes (Ohman et al., 2008).

**Figure 1:**
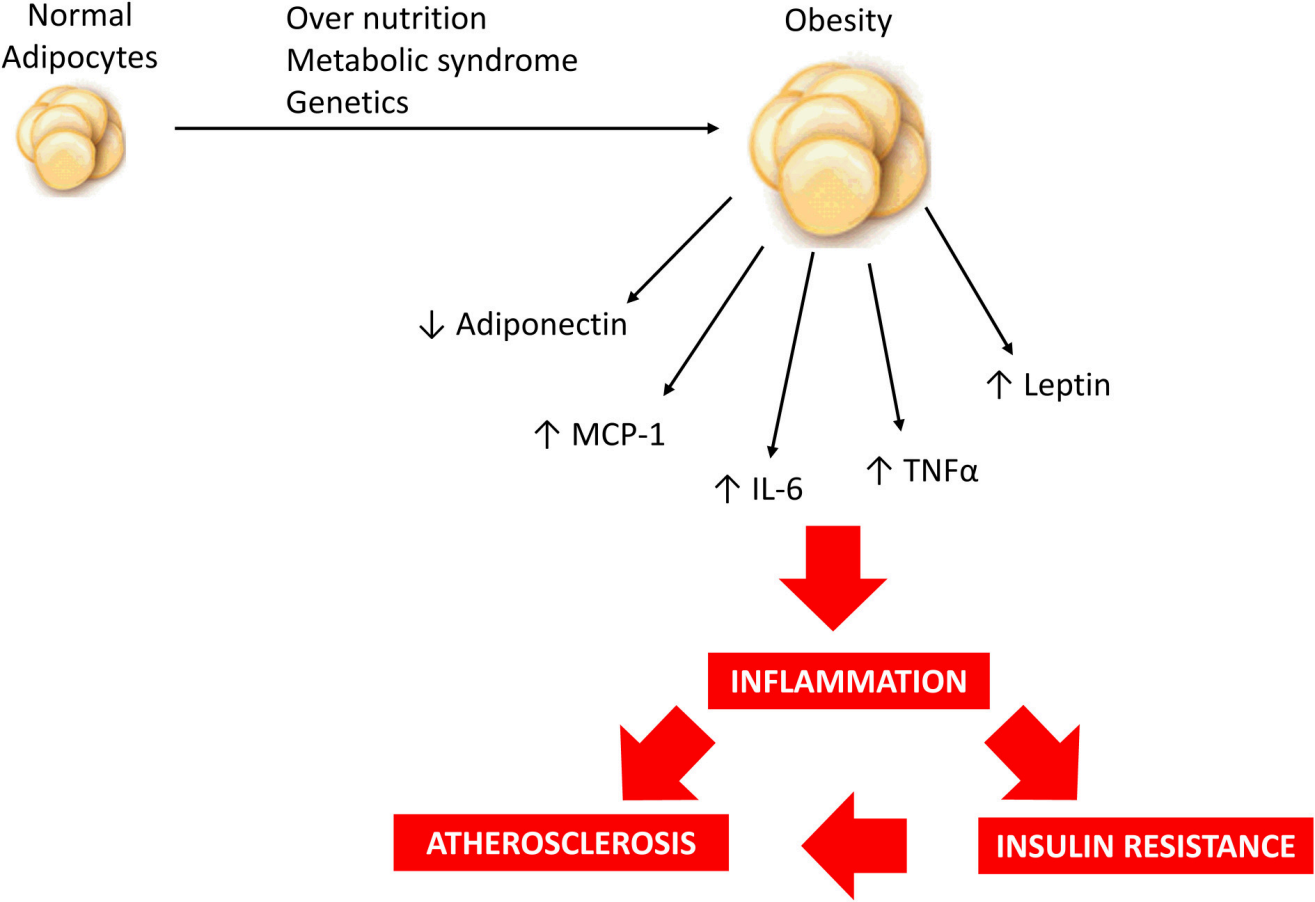
**Over nutrition, metabolic syndrome and/or genetic predisposition may contribute to obesity development and modulate adipokine profile resulting in a low-grade inflammatory state which is associated with increased risk of insulin resistance and atherosclerosis**.

Adipokines can be defined as a group of over 600 bioactive molecules produced by adipose tissue that acts as paracrine and endocrine hormones (Blüher, 2014). These molecules are important in the regulation of diverse processes including appetite and satiety, fat distribution, inflammation, blood pressure, hemostasis and endothelial function. They act in different organs including adipose tissue itself, brain, liver, muscle and vessels (Blüher, 2009, 2014; Lehr et al., 2012; Van de Voorde et al., 2013). These adipokines include mainly adiponectin, leptin, tumor necrosis factor alpha (TNFα), osteoprotegerin, interleukin 6 (IL-6), resistin, interleukin 1 (IL-1), apelin, visfatin, monocyte chemotactic protein-1 (MCP-1), plasminogen activator inhibitor-1 (PAI-1), retinol binding protein 4 (RBP4) and others (Van de Voorde et al., 2013; Blüher, 2014; Fisman and Tenenbaum, 2014). The pattern of secretion of adipokines can reflect adipose tissue function and this pattern is important to establish the individual risk to develop metabolic and cardiovascular comorbidities of obesity (Blüher, 2009, 2014). When adipose tissue inflammation and dysfunction are established, adipokine secretion is significantly changed toward a diabetogenic, proinflammatory and atherogenic pattern (Blüher, 2009, 2014), as represented in Figure 1. The nature of obesity-induced inflammation is different from other inflammatory situations such as infections or autoimmune diseases. Considering that obesity is a chronic condition, it produces a low-grade activation of innate immune system that affects homeostasis over time (Lumeng and Saltiel, 2011). It is important to highlight that adipose tissue macrophages (ATM) can also be considered as important sources of proinflammatory cytokines (Xu et al., 2015).

Although diabetes and other cardiovascular complications such as atherosclerosis have increasing importance in modern societies, the high mortality due to these diseases reveals that there are insufficient treatment options. Considering that, in the present review we provide an overview on the involvement of the main pro and anti-inflammatory adipokines in diabetes and atherosclerosis and discuss the therapeutic alternatives that could arise from the manipulation of these signaling pathways.

## Adipokines and diabetes

**Figure 2:**
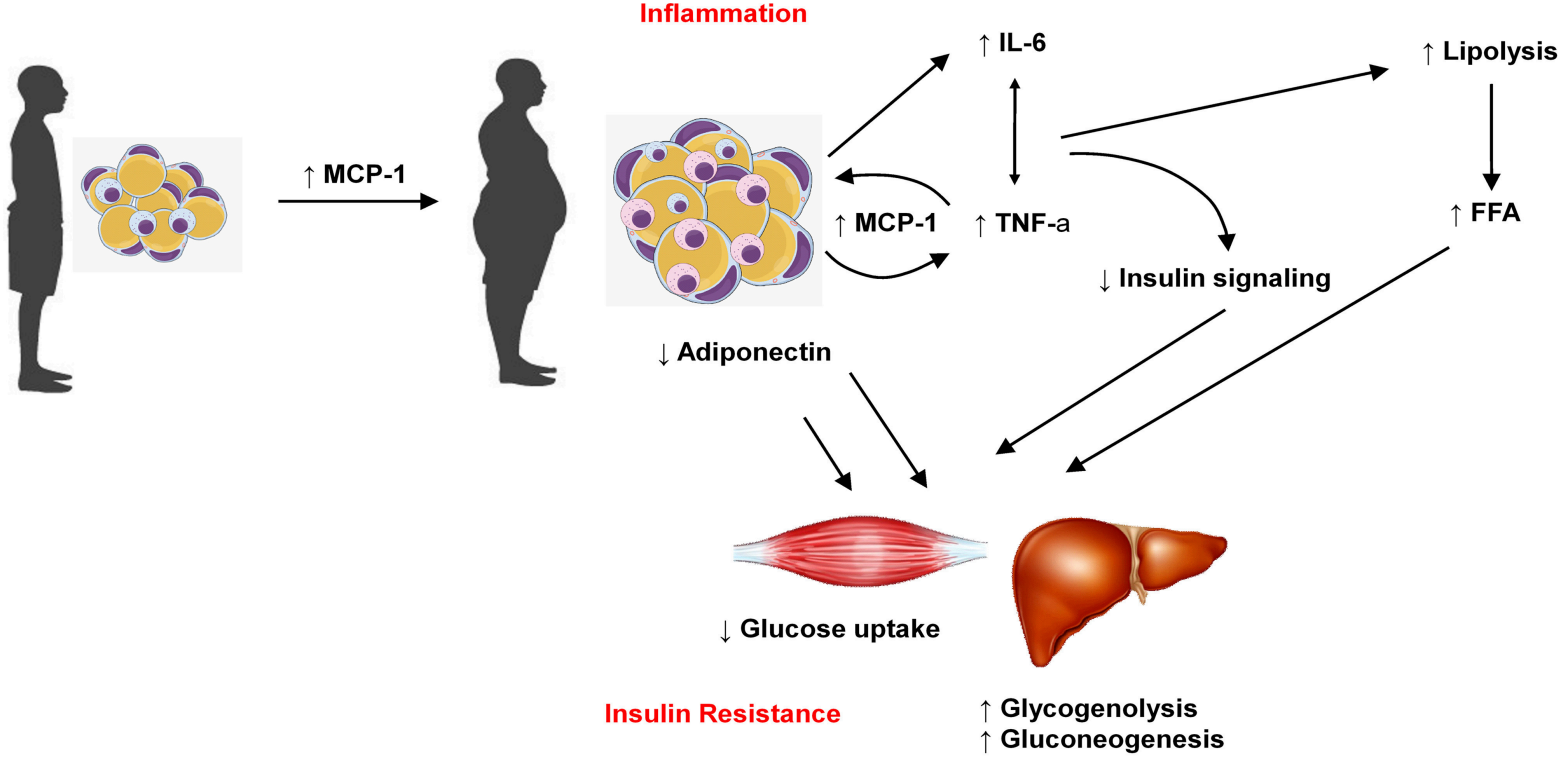
**Increase in adipose tissue mass is related to increase in MCP-1, which favors macrophages migration to adipose tissue and the production of other adipokines such as IL-6 and TNFα**. On the other hand, obese adipose tissue produces less adiponectin. Taken together, these features reduce glucose uptake in muscle cells and favors glycogenolisis and gluconeogenesis in liver, which contribute to insulin resistance and diabetes.

Insulin resistance (IR) is known to be an important factor underlying the pathogenesis of type 2 diabetes and it usually precedes the onset of this disease (Xu et al., 2015). It occurs in several tissues including liver, muscle and adipose tissue (Lee and Lee, 2014). Cytokines released by adipose tissue are involved in initiating and promoting a proinflammatory status, contributing to IR (Timar et al., 2014). Moreover, these molecules are involved in regulation of insulin sensitivity and secretion (Blüher, 2014). Thus, the impaired adipokine production observed in obesity contributes to diabetes pathogenesis. In metabolic syndrome (MS), adipocytes secrete factors that reduce insulin-mediated glucose uptake (including free fatty acids and proinflammatory cytokines) (Havel, 2002; Timar et al., 2014). The interaction if the main adipokines discussed in the present work to promote insulin resistance are illustrated in Figure 2.

### Modulation of insulin sensitivity by adiponectin

Adiponectin links visceral adiposity, IR, and atherosclerosis (Swarbrick and Havel, 2008). Unlike other adipokines, circulating concentration of adiponectin is inversely proportional to adiposity and low adiponectin levels predict the development of DM and CVD (Arita et al., 1999; Kadowaki et al., 2006). Moreover, strategies known to help delay or prevent DM and CVD like low-calorie, high-unsaturated fat diet and/or exercise are associated with increased circulating adiponectin levels (Esposito et al., 2003; Lim et al., 2014). Thus, adiponectin may contribute to the prevention of these diseases (Lim et al., 2014), although its use as a marker of cardiovascular risk is still controversial, as further discussed. Adiponectin production is primarily determined by adipocyte size and insulin sensitivity. It was observed that larger and insulin-resistant adipocytes produce less adiponectin (Bahceci et al., 2007; Swarbrick and Havel, 2008). Other cells and tissues can also secrete adiponectin, including cardiomyocytes (Waki et al., 2005; Huang et al., 2009; Caselli et al., 2014).

Independent groups revealed the insulin-sensitizing effect of adiponectin (Berg et al., 2001; Yamauchi et al., 2001; Kadowaki et al., 2006; Wascher et al., 2011). These researchers took the first steps to unravel the mechanisms involving adipokines and insulin resistance. Yamauchi et al. observed that the replenishment of adiponectin significantly ameliorates high-fat diet-induced insulin resistance and hypertriglyceridemia. They proposed that adiponectin is an insulin-sensitizing adipokine (Yamauchi et al., 2001). It has been described that an acute increase in the concentration of circulating adiponectin triggers a transient decrease in basal glucose level by inhibiting both the expression of hepatic gluconeogenic enzymes and the rate of endogenous glucose production in both wild-type and type 2 diabetic mice, suggesting that adiponectin sensitizes the body to insulin (Berg et al., 2001; Wascher et al., 2011). It was reported that a proteolytic cleavage product of adiponectin, which structurally resembles globular adiponectin, increases fatty-acid oxidation in muscle, decreases plasma glucose and causes weight loss in mice (Fruebis et al., 2001).

#### Adiponectin receptors

Adiponectin interacts with two different transmembrane receptors: AdipoR1 (expressed ubiquitously and at a high level in skeletal muscle) and AdipoR2 (expressed predominantly in the liver) (Yamauchi et al., 2003a; Scheid and Sweeney, 2014). It was detected that the deletion of both AdipoR1 and AdipoR2 in mice led to increased lipid accumulation in various tissues, IR and glucose intolerance. AdipoR1 deletion results in lack of adiponectin-stimulated adenosine monophosphate-activated protein kinase (AMPK) activation (Yamauchi et al., 2007). When AdipoR2 is deleted, the principal signaling defect occurs in peroxisome proliferator-activated receptor alpha (PPARα) signaling (Yamauchi et al., 2007) AdipoR1^−∕−^ mice exhibited decreased glucose tolerance and defective AMPK activation (Scheid and Sweeney, 2014). On the other hand, cultured myotubes from obese diabetic participants showed increased levels of AdipoR1 relative to lean controls (Holmes et al., 2011). Jang and colleagues reported that AdipoR2 levels are significantly lower in DM participants than in lean controls. These findings suggest that circulating levels of adiponectin and expression of AdipoR genes play an important role in the regulation of skeletal muscle insulin action (Chen et al., 2005; Jang et al., 2008; Holmes et al., 2011), although the exact effect of adiponectin in type 1 or type 2 receptors in different CVD is still under investigation. Okada-Iwabu and collaborators observed that orally active AdipoR agonists (AdipoRON) presented similar effects to adiponectin via AdipoR1 and 2 in both liver and skeletal muscle of diabetic mouse model, suggesting that adiponectin receptors could be a promising therapeutic target for the oral treatment of DM (Okada-Iwabu et al., 2013). Adiponectin receptors are also expressed in pancreatic β-cells and their expression is increased by exposure to free fatty acids, suggesting that adiponectin and its receptors are also involved in insulin secretion (Lim et al., 2014).

It is already established that AdipoR1 activates AMPK, promoting glucose uptake in muscle cells via translocation of GLUT4 transporters to cellular membrane (Fisman and Tenenbaum, 2014). Simultaneously, it blocks gluconeogenesis by inhibiting the hepatic enzyme phosphoenolpyruvate carboxylase, inhibits the synthesis of fatty acids and stimulates their oxidation (Kadowaki et al., 2006; Scheid and Sweeney, 2014). Moreover, adiponectin also increases fatty-acid combustion and energy consumption via activation of AdipoR2 through PPARα and PPARγ activation, which leads to glucose uptake and decreased triglyceride content in the liver and skeletal muscle, contributing to *in vivo* insulin sensitivity (Kumada et al., 2004; Kadowaki et al., 2006; Lim et al., 2014). Interestingly, AMPK can directly increase insulin sensitivity by stimulating the phosphorylation of peroxisome proliferator-activated receptor-γ co-activator 1 α (PGC-1α), a transcription co-activator that plays a critical role in the biosynthesis of mitochondria and oxidative phosphorylation. This reveals a cross-talk between the two different adiponectin receptors (Jäger et al., 2007; Scheid and Sweeney, 2014). Finally, adiponectin also enhances insulin sensitivity indirectly by increasing hepatic insulin receptor substrate 1 (IRS-1) expression via a macrophage-derived IL-6-dependent pathway. Thus, these multiple actions confer to adiponectin a key role in ensuring an effective protection against the development of IR (Awazawa et al., 2011; Fisman and Tenenbaum, 2014). In summary, adiponectin has insulin-sensitizing and cardiovascular-protective effects. These properties may help explain the inverse association between circulating adiponectin level and CVD, DM and obesity.

### TNFα-induced insulin resistance

The mechanistic link between obesity, DM, and adipose tissue inflammation was first proposed based on the finding that the level of the proinflammatory adipokine TNFα was increased in adipose tissue of obese rodents and humans and that its blockage led to improvement in insulin sensitivity (Hotamisligil et al., 1993). Subsequently, macrophages were found to infiltrate into adipose tissue of obese mice and humans and nearly 40–50% of total cells are F4/80-expressing macrophages in mice. These cells were also the major source of TNFα in adipose tissue (Weisberg et al., 2003; Cildir et al., 2013). The interaction between TNFα and its receptors, TNFR1 and TNFR2, mediates apoptosis, IR, lipolysis, inhibition of insulin-stimulated glucose transport and inhibition of insulin receptor autophosphorylation (Blüher, 2009; Cildir et al., 2013; Palomer et al., 2013). In adipocytes, TNFα reduces the secretion of adiponectin, induces IR and favors atherogenic dyslipidemia due to the reduction in GLUT4 expression, reduction in lipoprotein lipase (LPL) activity and increasing in expression of hormone-sensitive lipase (Cildir et al., 2013). TNFα impairs insulin signaling in adipocytes and hepatocytes through activation of stress-related protein kinases, as JNK-1, and activation of the IKKB/NF-κB pathway (Hirosumi et al., 2002; Arkan et al., 2005; Tarantino and Caputi, 2011) In addition, TNFα stimulates inhibitory phosphorylation of the serine residues of IRS-1, which is recognized as the major pathway in IR, corroborating the link between inflammation, obesity and IR (Wellen and Hotamisligil, 2005).

TNFα antagonism is efficient to treat patients with chronic inflammatory conditions such as rheumatoid arthritis. However, studies using anti-TNF therapies did not show significant improvement in insulin sensitivity. In obese Zucker rats, anti-TNF treatment had no effect on insulin sensitivity or lipid profile (López-Soriano et al., 1997; Cildir et al., 2013). Controversially, in some rodent studies, administration of TNFα antibodies resulted in inhibited inflammatory activity, improved fatty liver disease, protection against diet-induced obesity and IR (Li et al., 2003; Liang et al., 2008; Blüher, 2009). Unfortunately, the promising results from some animal studies using anti-TNF treatment in metabolic diseases were not successful clinically (Blüher, 2009). In a recent study, chronic TNFα neutralization by infliximab led to improvement in inflammatory status but did not ameliorate IR nor endothelial function in insulin-resistant volunteers (Wascher et al., 2011). Therefore, the effect of TNFα neutralization on insulin sensitivity in patients with DM needs to be further evaluated to open the perspective of new pharmacological targets.

### Interleukin-6 and insulin resistance

Interleukin-6 (IL-6) can be considered an adipokine since it is released by adipocytes from obese individuals, which occurs in a size-dependent manner (i.e., larger adipocytes release greater amounts of IL-6) and links obesity to a state of low-grade inflammation (Skurk et al., 2007; Schuett et al., 2009). The IL-6 family includes a range of diverse molecules such as IL-6 itself, IL-11, IL-27, IL-31, and others (Schuett et al., 2009; Scheller et al., 2011). These molecules have a common feature of containing two signal transducing receptor subunits, which one of them is glycoprotein 130 (gp130) (Schuett et al., 2009; Rose-John et al., 2015). Interestingly, only a few defined cell types (e.g., hepatocytes, monocytes, neutrophils and inactive T- and B-lymphocytes) express the specific IL-6 receptor (IL-6R) and can, therefore, respond to IL-6 classic signaling pathways (Schuett et al., 2009). However, a soluble form of IL-6R (sIL-6R) can be released due to shedding by A disintegrin and metalloprotease 17 (ADAM17). Interleukin-6 can bind to sIL-6R and activate gp130 in cells which do not former express IL-6R, a process known as trans-signaling (Matthews et al., 2003; Schuett et al., 2009; Scheller et al., 2011). This explains the wide range of effects elicited by this adipokine in different cell types. The exact metabolic role of IL-6 is still controversial because it has multiple functions, including tissue-specific effects on glucose metabolism and insulin signaling (Sabio and Davis, 2010). It appears to present dual functions, depending on the target tissue (liver or muscle), the duration of stimulus (acute vs. chronic) or the source of the cytokine (adipose tissue or skeletal muscle) (Schuett et al., 2009; Piya et al., 2013).

Chronically-elevated IL-6 has been described to be related to metabolic disorders such as obesity and IR (Franckhauser et al., 2008; Schuett et al., 2009). Circulating levels of IL-6 have been reported to be positively associated with MS, IR and diabetes (Ferreira-Hermosillo et al., 2015; Sindhu et al., 2015). Also, IL-6 mediates, at least in part, hepatic insulin resistance due to impairment of insulin receptor and IRS-1 phosphorylation (Sabio and Davis, 2010; Piya et al., 2013). It appears that the effect of IL-6 in hepatic control of insulin sensitivity and glucose tolerance is mediated by IL-6 classic rather than trans-signaling pathway (Scheller et al., 2011). This idea is reinforced by the fact that liver expresses IL-6R and that a long-term IL-6 trans-signaling inhibition in mice revealed no unfavorable metabolic effects (Schuett et al., 2009). Chronically-elevated IL-6 levels also lead to impaired insulin-mediated glucose uptake by muscle cells (Hassan et al., 2014). On the other hand, during exercise, acutely elevated IL-6 produced by skeletal muscle increases glucose uptake and AMPK-mediated fatty acid oxidation in these cells (Schuett et al., 2009; Piya et al., 2013). Considering the dual roles played by IL-6 on insulin sensitivity in diverse tissues and that these effects depend on different times of exposition and signaling pathways, further studies are necessary to ensure the safety of blocking IL-6 pathways as a pharmacological target to treat diabetes.

### MCP-1 and insulin resistance

Monocyte chemoattractant protein 1 (MCP-1) (also referred as chemokine C-C motif ligand 2, CCL2) is involved in leucocyte recruitment to inflammation sites. The effects of MCP-1 in recruiting monocytes, T lymphocytes and natural killer cells are dependent of the C-C motif chemokine receptor (CCR2), since the use of a specific antagonist for this receptor attenuates obesity-induced macrophage accumulation (Charo and Taubman, 2004; Weisberg et al., 2006; Gonzalez-Quesada and Frangogiannis, 2009). The interaction of MCP-1 with its receptor, CCR2, is considered pivotal for the recruitment of adipose tissue macrophages (ATMs) and the development of obesity-induced insulin resistance, although ATM recruitment can occur independently from MCP-1/CCR2 signaling (Xu et al., 2015). Adipocytes are an important source of MCP-1 and it causes adipose tissue inflammation even in the absence of macrophages (Sindhu et al., 2015). Adipose-derived MCP-1 is critical in exacerbating insulin resistance in adipose tissue of obese individuals (Uchida et al., 2012).

Interestingly, although chronic stress leads to atrophy of adipose tissue with a reduction in cell size, it also induces a low-grade inflammation status similar to obesity-related phenotype. In this model, MCP-1 is involved in the establishment of IR and a prothrombotic state (Uchida et al., 2012). Mice deficient in MCP-1 or the CCR2 are protected against high fat diet-induced IR (Weisberg et al., 2006). Although it was demonstrated that MCP-1/CCR2 signaling is important for obesity-induced insulin resistance and that the use of a CCR2 antagonist can ameliorate this condition without affecting macrophage infiltration into adipose tissue, it is difficult to state if IR is induced by MCP-1 *per se* or if it depends on recruited macrophages that release other cytokines (Kanda et al., 2006; Panee, 2012). Monocytes recruited into adipose tissue by CCR2 activation also secrete TNFα, IL-6 and MCP-1, which enhances the amplification cascade and favors continuous adipose tissue inflammation and IR through autocrine and paracrine interactions between monocytes and adipocytes (Uchida et al., 2012). Nevertheless, it is clear that MCP-1 links obesity to IR and macrophage infiltration into adipose tissue (Kanda et al., 2006). Importantly, MCP-1 is involved in diabetic nephropathy. Under stimulation of a high glucose concentration, advanced glycation end-products, oxidatively modified lipoproteins and angiotensin II, MCP-1 is expressed in mesangial cells, leading to glomerulosclerosis (Yadav et al., 2010).

### Leptin and insulin resistance

The implications of leptin in cardiovascular diseases has been studied since its first description in the classical paper by Zhang et al. (1994). Leptin is an adipose tissue-specific adipokine, known as a key molecule that regulates appetite, energy expenditure, behavior and glucose metabolism (Amitani et al., 2013; Adya et al., 2015). It crosses blood-brain barrier and, in hypothalamus, it acts in specific receptors to decrease appetite and increase energy expenditure (Koh et al., 2012). Also, it inhibits neuropeptide Y neurons (Elmquist et al., 1999). Leptin plasma concentration increases in proportion to body fat mass (Amitani et al., 2013). This adipokine acts on target cells through transmembrane receptors, which exist in 6 different isoforms (from Ob-Ra to Ob-Rf) (Koh et al., 2008; Adya et al., 2015). In obesity, despite increased leptin levels, a dysregulation of energy balance is observed, suggesting that obese people are resistant to leptin (Seufert et al., 2004; Koh et al., 2008). According to the concept of selective leptin resistance introduced by Mark and colleagues in 2002, only the anorectic effect of leptin is imbalanced, whereas other activities are maintained (Mark et al., 2002; Koh et al., 2012). It is important to highlight that exogenous leptin is efficient in promoting weight loss in obese humans and mice genetic deficient in leptin but not in diet-induced obesity (Blüher, 2014).

**Figure 3:**
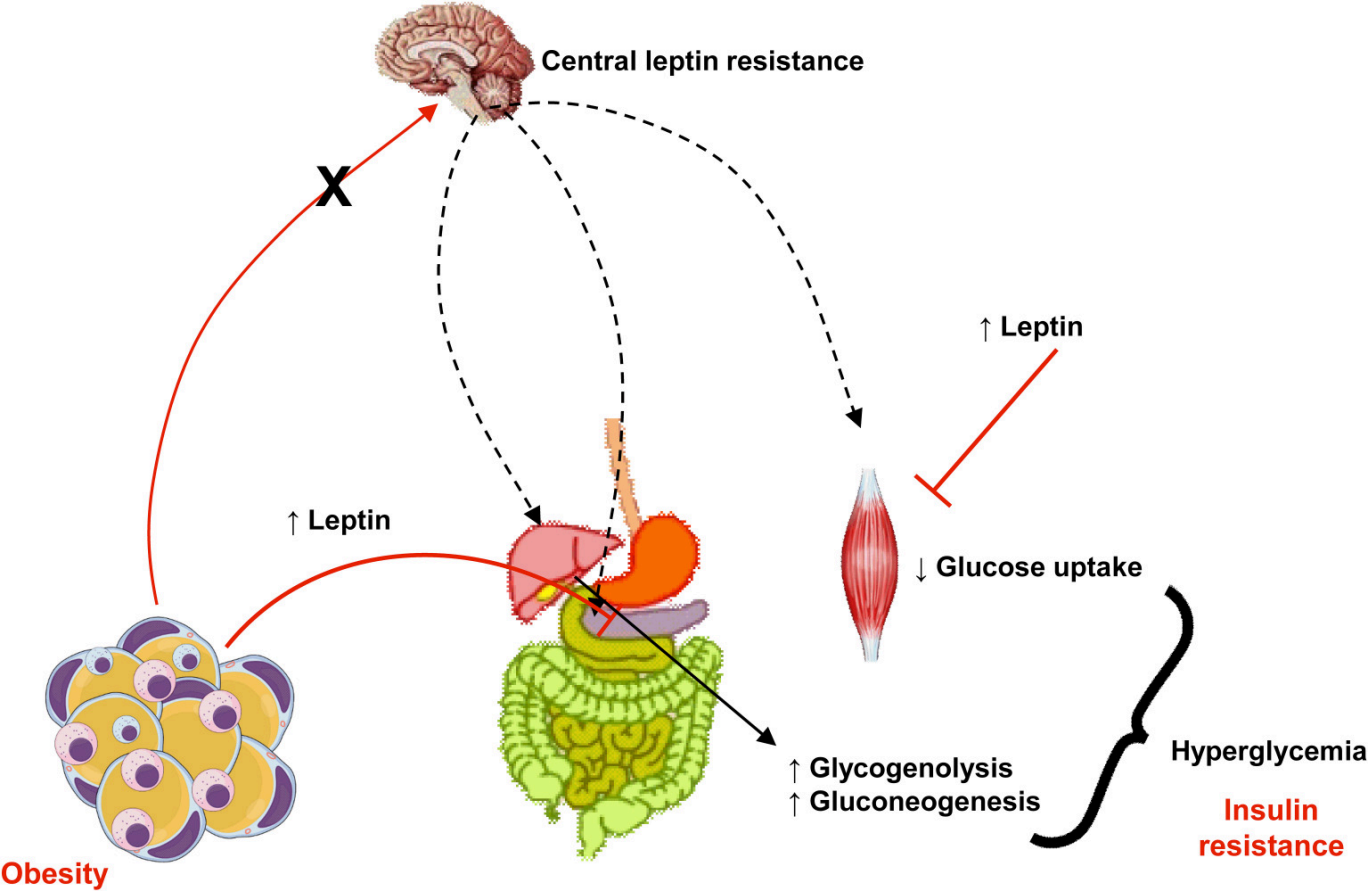
**In obesity, despite increased levels of leptin, central actions of this adipokine that control appetite, body weight, and energy expenditure are impaired (a phenomena known as leptin resistance, represented in the figure by the crossed red arrow from adipose tissue to the brain and black arrows)**. Otherwise, peripheral actions of leptin to reduce insulin secretion in pancreas and its signaling pathways is muscle (represented in the figure by plain arrows from adipose tissue to pancreas and muscle) culminates in insulin resistance, hyperglycemia and diabetes.

Leptin exerts an important role in regulation of glucose homeostasis, independent of its actions on food intake or body weight (Jung and Choi, 2014). Pancreatic β cells express leptin receptors and leptin inhibits insulin biosynthesis and secretion (Figure 3). There is a feedback loop where insulin stimulates leptin secretion from adipose tissue (Amitani et al., 2013) and leptin is decreased in low insulin states (Ahima and Flier, 2000). Several pathways are involved in leptin-induced inhibition of insulin secretion: suppression of preproinsulin mRNA, inhibition of GLP-1-induced insulin production, impairment of glucose transport via GLUT 2, regulation of ATP-sensitive potassium channels, inhibition of cAMP/PKA pathway, which regulates calcium channels and exocytosis (Seufert et al., 2004; Marroquí et al., 2012; Amitani et al., 2013). In skeletal muscle, leptin can impair GLUT 4 translocation, which contributes to insulin resistance (Figure 3) (Thorp and Schlaich, 2015). It was demonstrated that insulin resistance is associated with elevated plasma leptin levels (Segal et al., 1996). Thus, hyperleptinemia can be considering another critical link between obesity and insulin resistance. Given that leptin levels are increased in obesity and that, due to selective leptin resistance, its proinflammatory and insulin desensitizing effects are maintained, body weight reduction is important in diabetic patients as a strategy to preserve insulin efficacy.

## Adipokines and atherosclerosis

**Figure 4:**
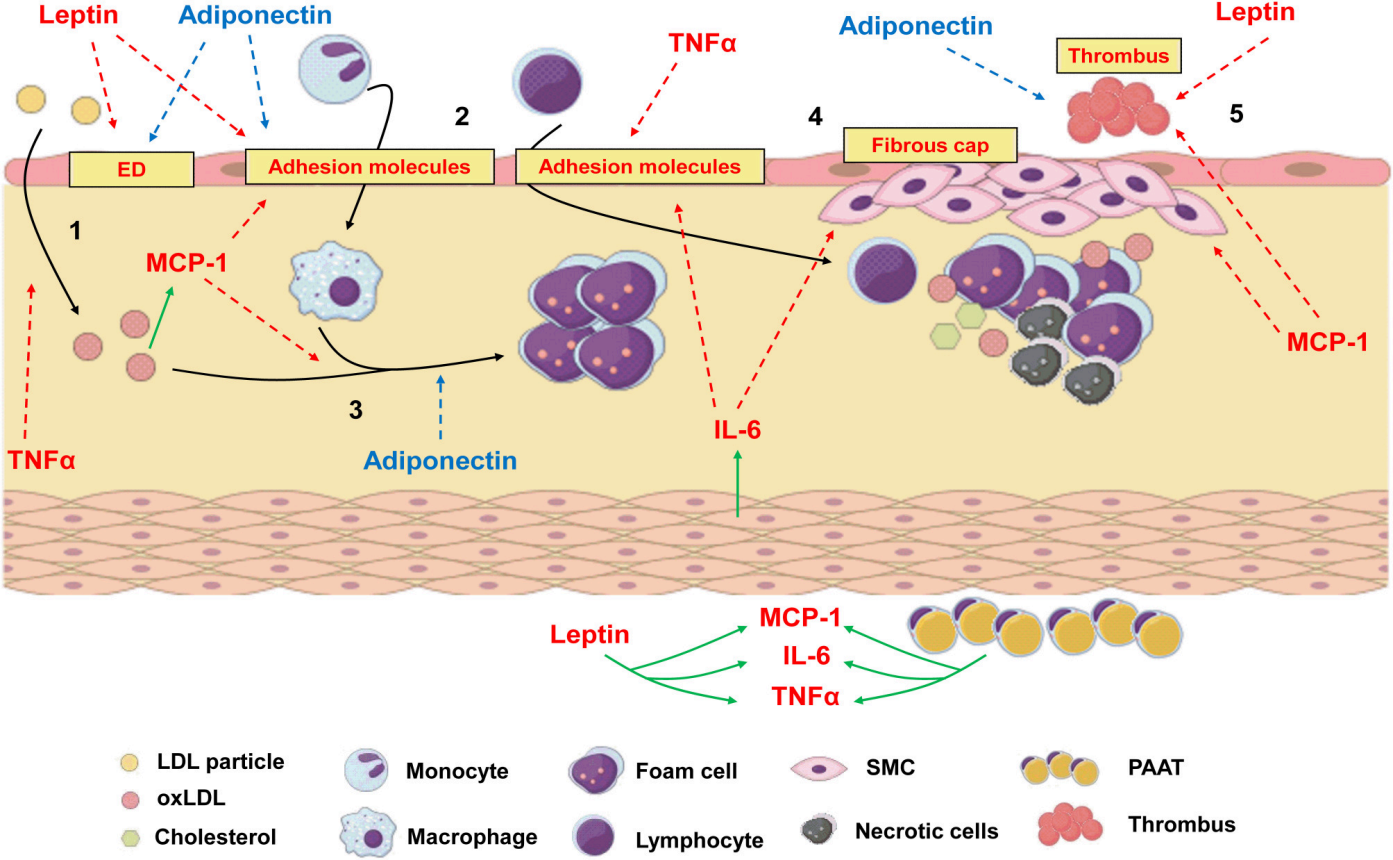
**The process of atherogenesis can be influenced by diverse adipokines.1:** Endothelial dysfunction (ED) and transmigration of LDL particles to subendothelial space can be worsen by leptin and TNFα. Adiponectin, which is reduced in obesity, recovers endothelial function. **2:** Once in subendothelial space LDL is oxidized (oxLDL), which is positively related to MCP-1 levels. Leptin, IL-6, MCP-1 and TNFα increase the expression of adhesion molecules in endothelium and increase leucocyte transmigration. **3:** Monocytes turn into macrophages under the stimulus of MCP-1 and phagocytes oxLDL, turning into foam cells. Adiponectin inhibits phagocytosis of oxLDL and foam cell formation. **4**: IL-6 can be produced by local smooth muscle cells (SMC) under the stimulus of angiotensin II. Along with MCP-1, it increases recruitment and proliferation of SMC and extracellular matrix deposition to form a fibrous cap around a lipid-rich necrotic core. **5**: Due to stimulation of matrix metalloproteinases and prothrombotic molecules, MCP-1 and leptin favors plaque rupture and thrombus formation, while adiponectin inhibits thrombosis. Periadventitial adipose tissue (PAAT) and leptin induce the production of proinflammatory adipokines. Red arrows represent proinflammatory pathways which are stimulated during obesity and contribute to atherogenesis. Blue arrows represent anti-inflammatory pathways which are inhibited during obesity since adiponectin levels are low. Green arrows represent production or stimulation of adipokine secretion.

Atherosclerosis can be defined as a chronic and progressive disease characterized by an inflammatory response of arterial wall to injuries promoted by risk factors such as dyslipidemia, diabetes, hypertension and others (Ross, 1999). The concept that atherosclerosis is an inflammatory disease is not new, since the inflammatory nature of atherosclerotic plaque was already described by Virchow in 1858 (Virchow, 1858, 1989). Although hypercholesterolemia figures among the most important risk factors for atherogenesis, nowadays it is well established that atherosclerosis is not only the accumulation of fat in arterial walls but is also a complex process involving both innate and adaptive immune processes (Ross, 1999; Hansson et al., 2002). In brief, atherogenic process initiates in sites where endothelium is submitted to shear stress (i.e., aortic root, aortic arch, superior mesenteric artery, and renal arteries). In these sites, endothelial dysfunction is observed and the permeability of the intimal layer is altered, favoring the migration of low density lipoprotein particles (LDL) to sub endothelial space (Tabas et al., 2007). Once endothelium has been activated by risk factors, it expresses adhesion molecules such as E-selectin, vascular cell adhesion molecule (VCAM-1) and intercellular adhesion molecule (ICAM-1), which attracts leukocytes. They adhere to endothelial lumen and migrate through vascular wall to the media. There, these cells express scavenger receptors and phagocyte oxidized LDL (oxLDL) turning into foam cells (Stephen et al., 2010). Growth factors and cytokines released by inflammatory cells contribute to the formation of a fibrous cap of smooth muscle and extracellular matrix around the lipid core, which compromises vascular lumen (Ross, 1999; Lusis, 2000; Hansson et al., 2002; Libby et al., 2010). Thus, it is clear that any factor involved in modulation of inflammatory response can influence atheroma development. Inflammatory process is not only involved in progression of atherosclerosis but is also responsible for acute thrombotic complications due to plaque rupture (Kumada et al., 2004), which may represent the major problematic event associated with atherosclerosis. Many adipokines can induce angiogenesis, which has deleterious effects on atheroma as the proliferation and migration of endothelial cells can lead to plaque destabilization and rupture (Van de Voorde et al., 2013). The adipokines effects on atherogenesis are illustrated in Figure 4.

### Adiponectin and atherosclerosis

Adiponectin can influence several steps in atheroma formation, from endothelial dysfunction to plaque rupture (Zhu et al., 2008; Lindgren et al., 2013). Accordingly to the “response to injury” theory of atherogenesis proposed by Ross (1999), the first step toward atheroma formation is an injury to endothelial wall. Considering that adiponectin can diminish endothelial response to mechanical injury (Fisman and Tenenbaum, 2014), it is clear that this adipokine present protective role in atherosclerosis. Hypercholesterolemia, the main risk factor to atheroma formation, can reduce endothelial progenitor cells (EPC) number and function (Dussault et al., 2009). Adiponectin was shown to recover EPC number and function, favoring endothelial repair (Huang et al., 2011; Issan et al., 2012; Fisman and Tenenbaum, 2014).

It has already been shown that adiponectin can inhibit the expression of VCAM-1, ICAM-1 and E-selectin by the endothelium (Ouchi et al., 2000), the initial phase of leukocyte migration through arterial wall. Adiponectin can modulate macrophage phenotype from the activated macrophage to an anti-inflammatory phenotype (Ouchi et al., 1999; Kumada et al., 2004), inhibiting its transformation into foam cell. Moreover, it can also reduce intracellular cholesteryl ester content, suppress TNFα production and stimulate the production of IL-10, which present anti-inflammatory features (Ouchi et al., 1999). In 2001, Ouchi and colleagues demonstrated that adiponectin is capable of diminishing the expression of class A scavenger receptors in macrophages, resulting in inhibition of foam cell transformation. In addition, it induced cholesterol efflux from macrophages due to upregulation of ATP-binding cassette transporter (ABCA1) (Ouchi et al., 2001; Tsubakio-Yamamoto et al., 2008). It has been shown that adiponectin is capable to increase the expression of tissue inhibitor of metalloproteinase 1 (TIMP1), protecting against plaque rupture and thrombotic events (Kumada et al., 2004).

Although adiponectin can be considered as an anti-inflammatory adipokine, some studies have indicated that its level is related more closely to the degree of insulin resistance than to the degree of adiposity in humans (Weyer et al., 2001; Ohman et al., 2008) and that the relationship between adiponectin concentration and CVD is still controversial (Weyer et al., 2001). One possible explanation is the different forms of adiponectin found in plasma and their diverse biological effects. Adiponectin can be present as a trimer, with anti-inflammatory properties, as a trimer-dimer or as a large multimeric structure, which present proinflammatory effect. In this context, the percentage of the different isoforms observed in diverse pathophysiological conditions could be responsible for discrepant observations (Kim et al., 2012; Hao et al., 2013). Another possible explanation is that adiponectin acts differently depending on the receptor activated. This adipokine can mediate its effects via AdipoR1 and AdipoR2, respectively. In 2013, Lindgren and colleagues demonstrated that crossbreeding apolipoprotein E knockout mice (apoE^−∕−^) and AdipoR2^−∕−^ animals can generate a lineage which presents smaller plaque area in brachiocephalic artery, suggesting that the activation of this receptor has proatherogenic effect, despite no differences in plasma lipid profile (Lindgren et al., 2013). However, it was described that overexpression of adiponectin protects against atherosclerosis in apoE^−∕−^ mice (Yamauchi et al., 2003b). Some authors defend that adiponectin can be used as a marker of cardiovascular risk, once it correlates negatively with coronary artery disease (Ouchi et al., 1999), although the diverse actions of this adipokine and different responses depending on which receptor is activated make it still a controversial issue. Nevertheless, it is generally well accepted that hypoadiponectinemia (<4 μg/mL) is associated with a variety of diseases, including atherosclerosis, DM, hypertension and others, although hyperadiponectinemia can be associated with increased renal and pulmonary diseases (Kishida et al., 2014).

### TNFα and atherosclerosis

Although it was initially suggested that the main source of TNFα in obesity were adipocytes, it is now well recognized that M1 macrophages infiltrated in adipose tissue are responsible for increased levels of this cytokine (Arkan et al., 2005; Solinas et al., 2007; Galic et al., 2010; Nakamura et al., 2014). Still, TNFα was the first adipokine suggested to represent a link between obesity, inflammation and diabetes (Hotamisligil et al., 1993; Galic et al., 2010).

Similarly to adiponectin, TNFα is considered to be involved in all aspects regarding atheroma formation, although it presents proinflammatory properties. In endothelial cells, TNFα induces the activation of proinflammatory, procoagulant and proliferative genes (Ohta et al., 2005; Xiao et al., 2009; Ntaios et al., 2013; Nakamura et al., 2014; Steyers and Miller, 2014). Considering that endothelial dysfunction can be defined as an unbalance in the production of vasoconstrictors and vasodilators, pro and anti-inflammatory substances, inhibitor and stimulator factors and pro and anti-coagulators (Rubanyi, 1993), the production of TNFα during obesity can be considered as an inductor of endothelial dysfunction (Kobayasi et al., 2010; Steyers and Miller, 2014). Endothelial dysfunction is the first event in atherogenesis pathway (Ross, 1999) and the induction of this stat by TNFα is, at least in part, responsible for the increased incidence of atherosclerosis-related events in obese patients. Another marker of endothelial dysfunction is the inability of acetylcholine to induce endothelium-dependent relaxation in vessel preparations *in vitro* (Balarini et al., 2013) and this was also induced by TNFα (Wang et al., 1994) since it reduces endothelial nitric oxide synthase (eNOS) expression and activity (Steyers and Miller, 2014). Vasocrine signaling by TNFα derived from periadventitial adipose tissue (PAAT) is also responsible for decreased NO production and endothelial dysfunction (Yudkin et al., 2005; Ronti et al., 2006).

Another aspect of atherosclerosis-related inflammation that is induced by TNFα is the alteration in endothelial permeability. This adipokine can increase the expression of adhesion molecules (ICAM-1, VCAM-1, and E-selectin) by endothelium and alter endothelial cell morphology, augmenting its permeability not only to immune cells but also to small particles like LDL (Marcos-Ramiro et al., 2014; Steyers and Miller, 2014). Interestingly, Zhang and colleagues demonstrated that TNFα is capable to induce transcytosis of LDL at the first stages of atherosclerosis development through a mechanism dependent of nuclear factor kappa B (NF-κB) and peroxisome proliferator-activated receptor gamma (PPAR-γ) crosstalk (Zhang et al., 2014). The activation of endothelium and increasing in expression of adhesion molecules by TNFα can be reduced by adiponectin (Van de Voorde et al., 2013).

TNFα is first synthesized as a transmembrane protein and then is turned into is soluble form through the cleavage by ADAM-17 (A disintegrin A metalloproteinase 17), which increased activity is related to ischemia, heart failure, atherosclerosis, diabetes and hypertension (Peschon et al., 1998; Menghini et al., 2013; Xia et al., 2013; Speck et al., 2015). There is only one endogen inhibitor of ADAM-17, known as tissue inhibitor of metalloproteinase 3 (TIMP3), which activity is reduced in obesity, atherosclerosis, diabetes and insulin resistance (Chavey et al., 2003; Cardellini et al., 2009, 2011; Menghini et al., 2013). This results in increased ADAM17 action and augmented TNFα.

Adipokines can also be considered important clinical biomarkers and pharmacologic targets to treat atherosclerosis. Blood level of TNFα was associated with coronary heart disease in elderly, serving as a biomarker for CVD risk (Cesari et al., 2003). The inhibition of TNFα-induced signaling pathways that lead to LDL transcytosis was efficient in reducing atherosclerosis in experimental model (Zhang et al., 2014) as well as silencing of TNFα-encoding gene (Brånén et al., 2004). Apart from decreasing plasma cholesterol, simvastatin reduced leptin, and TNFα, increased adiponectin levels and decreased TNFα-induced apoptosis of endothelial progenitor cells (EPC), which contributes to clinical effectiveness of this class of drugs (Du et al., 2014; Krysiak et al., 2014). This highlights that strategies aimed to modulate inflammatory actions elicited by TNFα could be promising therapeutic options to treat atherosclerosis.

### IL-6 and atherosclerosis

Interleukin 6 (IL-6) is an important adipokin secreted by adipocytes. However, it can also be released by smooth muscle cells under the influence of angiotensin II (Libby, 2002). Tikellis and colleagues demonstrated that feeding apoE^−∕−^ mouse with a low salt diet activated the renin-angiotensin-aldosterone system (RAAS) and increased IL-6 in serum and aorta (Tikellis et al., 2012). As previously discussed, IL-6 can present different effects depending on the signaling pathway activated (classic or trans-signaling), the duration of the stimulus and the source of the cytokine (Schuett et al., 2009; Piya et al., 2013). It seems that pro- and anti-inflammatory effects of IL-6 depends on ADAM17 activation and the balance between the activation of classic and trans-signaling cascades (Scheller et al., 2011). Considering that in atherosclerosis ADAM17 is overactive (Speck et al., 2015), IL-6 is expected to present a proinflammatory role in this disease.

In endothelial cells, IL-6 trans-signaling is responsible for the up regulation of adhesion molecules such as ICAM-1, VCAM-1 and E-selectin and the control of lymphocytes trafficking (Chen et al., 2006; Scheller et al., 2011), which in turn favors an proatherogenic phenotype. Also, this adipokine contributes to the differentiation of monocytes into macrophages (Chomarat et al., 2000). Interestingly, Speck and colleagues observed that the antiatherogenic effect of fish oil is due to reduction in ADAM17 activity. The consequent decreased release of endothelial adhesion molecules would contribute to endothelial barrier improvement. Moreover, authors found a reduction in sIL-6R in animals that received fish oil (Speck et al., 2015), which reinforces the role played by IL-6 trans-signaling in early stages of atherosclerosis development. Additionally, IL-6 is negatively correlated with EPC number in patients with rheumatoid arthritis. These patients present increased morbidity and mortality attributable to accelerated atherosclerosis and develop endothelial dysfunction and EPC impaired function even at young age (Herbrig et al., 2006). IL-6 can also influence the production of other cytokines. It was reported that the increase in C-reactive protein (CRP), a marker of acute inflammation, is rather produced by the liver under the influence of IL-6 than a direct product of adipose cells (Bays, 2009). It was described that IL-6 can stimulate the production of matrix metalloproteinases, which contribute to plaque vulnerability/rupture and arterial remodeling (Watanabe and Ikeda, 2004; Schuett et al., 2009).

Blocking IL-6 effects using neutralizing monoclonal antibodies to treat atherosclerosis is controversial. In patients with lymphoproliferative disorder it was associated with an increase in body weight, hypertriglyceridemia and hypercholesterolemia (Nishimoto et al., 2005), which could favor the development of atherosclerotic plaques. Although inhibition of IL-6 with tocilizumab (an antibody that binds to both soluble and membrane bound IL-6R) has been reported to improve endothelial function and reduce arterial stiffness (Protogerou et al., 2011), it also increases LDL-cholesterol (Ridker and Lüscher, 2014). Thus, anti-IL-6 therapies are still considered a double-edged sword in atherosclerosis management. On the other hand, indirect approaches can reduce inflammatory actions of IL-6. Statin therapy was reported to reduce the IL-6-induced production of CRP and MCP-1, important inflammatory markers (Rodriguez et al., 2012). Blocking renin-angiotensin system (RAAS) with an inhibitor of angiotensin converting enzyme (ACE) reduced IL-6 and inflammation markers in atherosclerosis experimental model (Tikellis et al., 2012).

### MCP-1 and atherosclerosis

The release of MCP-1 by endothelial cells, smooth muscle cells, T cells, monocytes, macrophages and foam cells perpetuates inflammation and lipid accumulation in atheroma (Tabata et al., 2003; Lin et al., 2014), although experimentally this depends on a high cholesterol diet (Namiki et al., 2002). Also, adipocyte-derived MCP-1 is overexpressed in obesity, in proportion of adiposity (Weisberg et al., 2006). In early atheroma formation, MCP-1 can be considered the link between oxLDL and foam cell recruitment to vessel wall whereas oxLDL (but not native LDL) induce MCP-1 production (Cushing et al., 1990). Apart from migration of monocytes/macrophages, MCP-1 also controls its differentiation into foam cells. During this process, the number of LDL receptors decrease while the number of scavenger receptors (responsible for phagocytosis of oxLDL) increases (Stephen et al., 2010). It was reported that MCP-1 induce the expression of scavenger receptors on monocytes through extracellular signal-regulated kinase (ERK) (Tabata et al., 2003). In summary, MCP-1 can be considered a key molecule in the regulation of oxLDL phagocytosis/foam cell formation sequence. Interestingly, Hashizume and Mihara showed that oxLDL-induced MCP-1 was augmented by IL-6 and TNFα and that this mechanism is also involved in the induction of scavenger receptors by IL-6 and TNFα, which creates a self-perpetuating and amplifying cycle of inflammation and atherogenesis and suggests that IL-6 and TNFα participate in atherogenesis process also via oxLDL/MCP-1 induction (Hashizume and Mihara, 2012; Uchida et al., 2012). During fibrous cap formation around the lipid core, MCP-1 participates in smooth muscle cells (SMC) proliferation and activation (Gonzalez-Quesada and Frangogiannis, 2009).

MCP-1 is involved not only in the initial phase of atherosclerosis development but also in the final fatal complication of atherosclerotic plaque rupture and thrombosis. In endothelial cells, MCP-1 can induce the secretion of matrix metalloproteinases (Werle et al., 2002; Gonzalez-Quesada and Frangogiannis, 2009), which is crucial for plaque disruption. In addition, it was described that MCP-1 contributes to thrombin generation, thrombus formation and upregulation of tissue factor and plasminogen activation inhibitor-1 (PAI-1) (Charo and Taubman, 2004; Gonzalez-Quesada and Frangogiannis, 2009; Uchida et al., 2012).

Gonzalez-Quesada and Frangogiannis state that the effects of MCP-1 inhibition after myocardial infarction should be carefully evaluated because the suppression of this adipokine could delay the phagocytosis of dead cardiomyocytes and extend the injury extension (Gonzalez-Quesada and Frangogiannis, 2009). In this context, similarly to IL-6, indirect approaches that decrease not only MCP-1 but also other inflammatory markers are of interest. Statins were shown to decrease MCP-1, IL-6, IL-8 in hypercholesterolemic patients (Rezaie-Majd et al., 2002), although this might be dependent on treatment duration since that high doses of atorvastatin during 5 days did not modified inflammatory markers, including MCP-1, in aorta of high fat-feeding apoE^−∕−^ mice (Ekstrand et al., 2015). Nevertheless, MCP-1 inhibition resulted in decreased TNFα, IL-6, tissue factor and PAI-1 in an inflammation model induced by stress in mice (Uchida et al., 2012), suggesting a beneficial effect not only in inflammation but also in the prothrombotic state in the presence of atherosclerosis.

### Leptin and atherosclerosis

Unlike other adipokines, which are produced from different sources, leptin is mainly produced by adipocytes and plasma levels are positively correlated with white adipose tissue mass (Scotece et al., 2012). It regulates energy balance and metabolism both centrally and peripherally (Koh et al., 2012). In cardiovascular system, blood vessels and cardiomyocytes express the specific leptin receptor (named Ob-R, which presents 6 isoforms) and its actions are potentially proatherogenic, prothrombotic and angiogenic (Koh et al., 2012; Scotece et al., 2012; Adya et al., 2015). Interestingly, in obese individuals only the anorectic effect of leptin is impaired, whereas other effects are maintained (a phenomenon known as selective leptin resistance) (Mark et al., 2002; Singh et al., 2010; Adya et al., 2015) thus hyperleptinemia contributes to atherogenesis in these patients.

Hyperleptinemia is associated to impairment of NO-dependent vasorelaxation, increase in oxidative stress as well as increase in endothelin (a potent vasoconstrictor) (Yamagishi et al., 2001; Adya et al., 2015; Husain, 2015). All these features are markers of endothelial dysfunction, the first step in atherogenesis. It was described that leptin increases NADPH oxidase expression and activity (Dong et al., 2006; Schroeter et al., 2012). Moreover, it increases the expression of type-1 angiotensin II receptor (AT1R) in smooth muscle cells (Zeidan et al., 2005). It is well established that angiotensin II increases oxidative stress through AT1R-dependent activation of NADPH oxidase (Braga et al., 2011). Thus, leptin potentiates deleterious angiotensin II effects in vascular function, which is potentially dangerous in hypertensive obese patients. Conversely, angiotensin II increases leptin synthesis (Koh et al., 2012), generating a self-perpetuating cycle of excessive oxidative stress and vascular dysfunction.

In initial phase of atheroma formation, leptin plays a crucial role in inflammatory pathways. It increases the secretion of TNFα, IL-6, and MCP-1 (Yamagishi et al., 2001; Koh et al., 2012) important inflammatory molecules as previously discussed. The expression of adhesion molecules such as VCAM-1, ICAM-1, and E-selectin are also increased by leptin (Adya et al., 2015). This favors monocytes attraction and migration through endothelial wall. During atheroma formation, leptin is also involved growth and migration of SMC (Zeidan et al., 2005; Husain, 2015). Leptin also induces a prothrombotic state once it enhances platelets activation and aggregation, thrombus formation and PAI-1 expression (Beltowski, 2006; Singh et al., 2010; Husain, 2015). Plaque rupture is an important event that usually precedes thrombus formation and leptin is involved in plaque rupture since it induces the production of MMP (Li et al., 2005; Adya et al., 2015).

Hyperleptinemia is related to acute cardiovascular events independent of traditional risk factors (Koh et al., 2012). Leptin treatment was described to be efficient in reducing weight in leptin-deficient obese mice and humans, but this effect was small in diet-induced obesity (Blüher, 2014), probably due to the selective leptin resistance previously mentioned. Although leptin may be involved in inflammation response in certainly conditions, inhibition of inflammatory cytokines such as TNFα did not modified leptin levels (Scotece et al., 2012). On the other hand, considering the synergism between angiotensin II and leptin, antihypertensive therapies may decrease leptin levels. Umeda and colleagues demonstrated that inhibition of AT1R decreased leptin in adipose tissue (Umeda et al., 2003). Also, inhibition of angiotensin II synthesis decreased leptin (Cassis et al., 2004). These results highlight the importance of blood pressure control in obese hypertensive patients, especially using RAAS-antagonists.

### Periadventitial adipose tissue

Considering that patients with autoimmune diseases have increased risk for atherosclerosis, inflammation in different sites can be involved in atherogenesis, possibly due to the generation of cytokines and other factors that can be released into the circulation (Hahn et al., 2007; Rosenfeld, 2013). Medium and large arteries, where atherosclerotic plaques develop, are surrounded by periadventitial adipose tissue (PAAT), which provides chemical messengers and vasoactive mediators into the bloodstream and function as a paracrine organ (Mattu and Randeva, 2013; Chaldakov et al., 2014). Even though the view of atherosclerosis has been mainly focused on intimal lesions and luminal loss, it is likely that other components of vascular wall are involved in this inflammatory process (Chaldakov et al., 2014). In this regard, inflamed PAAT can be considered as an important source of pro and anti-inflammatory adipokines which contribute to plaque formation and stabilization. In the heart, adventitial lymphocytic inflammation is related to epicardial fat metabolism. It was observed that, in epicardial fat harvest during coronary bypass, there was an increase in proinflammatory markers (as IL-6, MCP-1, and TNFα) when compared to abdominal fat (Mazurek et al., 2003; Tavora et al., 2010). This confirms the relation between PAAT and atherosclerosis. Moreover, inflamed fat from other sources can also be responsible, at least in part, for atherogenesis. It was already shown that that inflammatory visceral fat accelerated atherosclerosis in apoE^−∕−^, possibly due to increase in MCP-1 since a pharmacological approach capable of reducing MCP-1 was efficient in reducing atherosclerosis in this model (Ohman et al., 2008).

## Conclusion

In summary, it is now well established that adipose tissue can be considered a source of diverse molecules, which play important roles in the body homeostasis. Obese adipose tissue can induce a state of low-grade inflammation due to secretion of proinflammatory adipokines and the reduced secretion of anti-inflammatory ones. In this brief review we highlighted the participation of the main adipokines in insulin resistance, diabetes and atherosclerosis. The comprehension of molecular pathways involved in the mechanism of action of these molecules created the possibility for clinical and translational studies aiming to provide new therapeutic interventions. Patients who suffer from chronic inflammatory diseases present increased risk of diabetes and atherosclerosis. However, the use of anti-inflammatory therapies to treat these conditions is still controversial and often the results are inferior to the expected. On the other hand, indirect approaches which culminate in reduction of adipokines secretion or signaling seems to be promising. Nevertheless, considering that obesity is a manipulable risk factor which is often related to individual life style, an important approach to prevent CVD and diabetes is still the alteration of bad alimentary habits and reduction in body weight.

## Author contributions

All authors participated in the design of the manuscript, drafted the manuscript, revised the manuscript critically and approved the final version.

### Conflict of interest statement

The authors declare that the research was conducted in the absence of any commercial or financial relationships that could be construed as a potential conflict of interest.
